# The ELISA Detectability and Potency of Pegfilgrastim Decrease in Physiological Conditions: Key Roles for Aggregation and Individual Variability

**DOI:** 10.1038/s41598-020-59346-z

**Published:** 2020-02-12

**Authors:** Tao Xie, Hui Fang, Weiming Ouyang, Phillip Angart, Meng-Jung Chiang, Ashwinkumar A. Bhirde, Faruk Sheikh, Patrick Lynch, Ankit B. Shah, Sharadrao M. Patil, Kang Chen, Meiyu Shen, Cyrus Agarabi, Raymond P. Donnelly, Kurt Brorson, Sarah J. Schrieber, Kristina E. Howard, Sarah M. Rogstad, David M. Frucht

**Affiliations:** 1 Office of Biotechnology Products, Office of Pharmaceutical Quality, Center for Drug Evaluation and Research, U.S. Food and Drug Administration, Silver Spring, Maryland United States of America; 2 Office of Clinical Pharmacology, Office of Translational Sciences, Center for Drug Evaluation and Research, U.S. Food and Drug Administration, Silver Spring, Maryland United States of America; 3 Office of Testing and Research, Office of Pharmaceutical Quality, Center for Drug Evaluation and Research, U.S. Food and Drug Administration, Silver Spring, Maryland United States of America; 4 Office of Biostatistics, Office of Translational Sciences, Center for Drug Evaluation and Research, U.S. Food and Drug Administration, Silver Spring, Maryland United States of America

**Keywords:** Recombinant protein therapy, Drug development

## Abstract

PEGylated recombinant human granulocyte colony stimulating factor (pegfilgrastim) is used clinically to accelerate immune reconstitution following chemotherapy and is being pursued for biosimilar development. One challenge to overcome in pegfilgrastim biosimilar development is establishing pharmacokinetic (PK) similarity, which is partly due to the degree of PK variability. We herein report that commercially available G-CSF and PEG ELISA detection kits have different capacities to detect pegfilgrastim aggregates that rapidly form *in vitro* in physiological conditions. These aggregates can be observed using SDS-PAGE, size-exclusion chromatography, dynamic light scattering, and real-time NMR analysis and are associated with decreased bioactivity as reflected by reduced drug-induced cellular proliferation and STAT3 phosphorylation. Furthermore, individual variability in the stability and detectability of pegfilgrastim in human sera is also observed. Pegfilgrastim levels display marked subject variability in sera from healthy donors incubated at 37 °C. The stability patterns of pegfilgrastim closely match the stability patterns of filgrastim, consistent with a key role for pegfilgrastim’s G-CSF moiety in driving formation of inactive aggregates. Taken together, our results indicate that individual variability and ELISA specificity for inactive aggregates are key factors to consider when designing and interpreting studies involving the measurement of serum pegfilgrastim concentrations.

## Introduction

The recombinant human granulocyte colony stimulating factor (G-CSF) product, filgrastim, and the related pegylated molecule, pegfilgrastim, have been licensed for the prevention of chemotherapy-induced neutropenia and its life-threatening complications. These drugs bind to cell surface G-CSF receptors (G-CSF-R) on mature neutrophils and their precursors, leading to increased cell division and expedited transit through the bone marrow to the peripheral blood and tissues^1,2^. Pegfilgrastim was developed by covalently linking a ~20 kDa polyethylene glycol (PEG) molecule to the N-terminus methionine residue of filgrastim. PEGylation of G-CSF increases the total molecular weight from 18.8 to 39 kDa, while maintaining the biological activity of G-CSF^3^. However, the biological half-life of pegfilgrastim is significantly longer^4^, allowing administration once per chemotherapy cycle^4–6^. The difference in half-life between filgrastim and pegfilgrastim has been attributed to the presence of the PEG moiety on pegfilgrastim, which, by increasing the hydrodynamic size, decreases renal clearance^4,7–9^. In contrast to filgrastim, pegfilgrastim clearance is hypothesized to be mediated via the neutrophil G-CSF-R^10^.

U.S.-licensed Neulasta (pegfilgrastim) has a large commercial market ($4.7 B in 2017)^11^ and has recently been the target of biosimilar development in the United States, where three biosimilar products to US-licensed Neulasta have been FDA-approved to date. Pharmacokinetic (PK) similarity is an essential component of establishing a biosimilar product. The serum levels of pegfilgrastim following treatment, which are commonly determined using enzyme-linked immunosorbent assays (ELISAs), show a high degree of human subject variability^12–16^. One interpretation of the results of these studies is that the time-dependent decline in the levels of detected pegfilgrastim reflects clearance of the drug via G-CSF-R-mediated pathways, but this conclusion assumes that pegfilgrastim retains a stable ELISA signal *in vivo*.

We herein report that pegfilgrastim aggregates rapidly in physiological conditions, leading to reduced potency and ELISA detectability. Moreover, we observe that there is marked individual variability in the stability and detectability of pegfilgrastim in human sera. These factors should be considered in the design (PK study sample size, dosing, sample handling, etc.) and interpretation of clinical studies involving the measurement of pegfilgrastim concentrations.

## Results

### Pegfilgrastim loses ELISA detectability in physiological conditions

**Figure 1:**
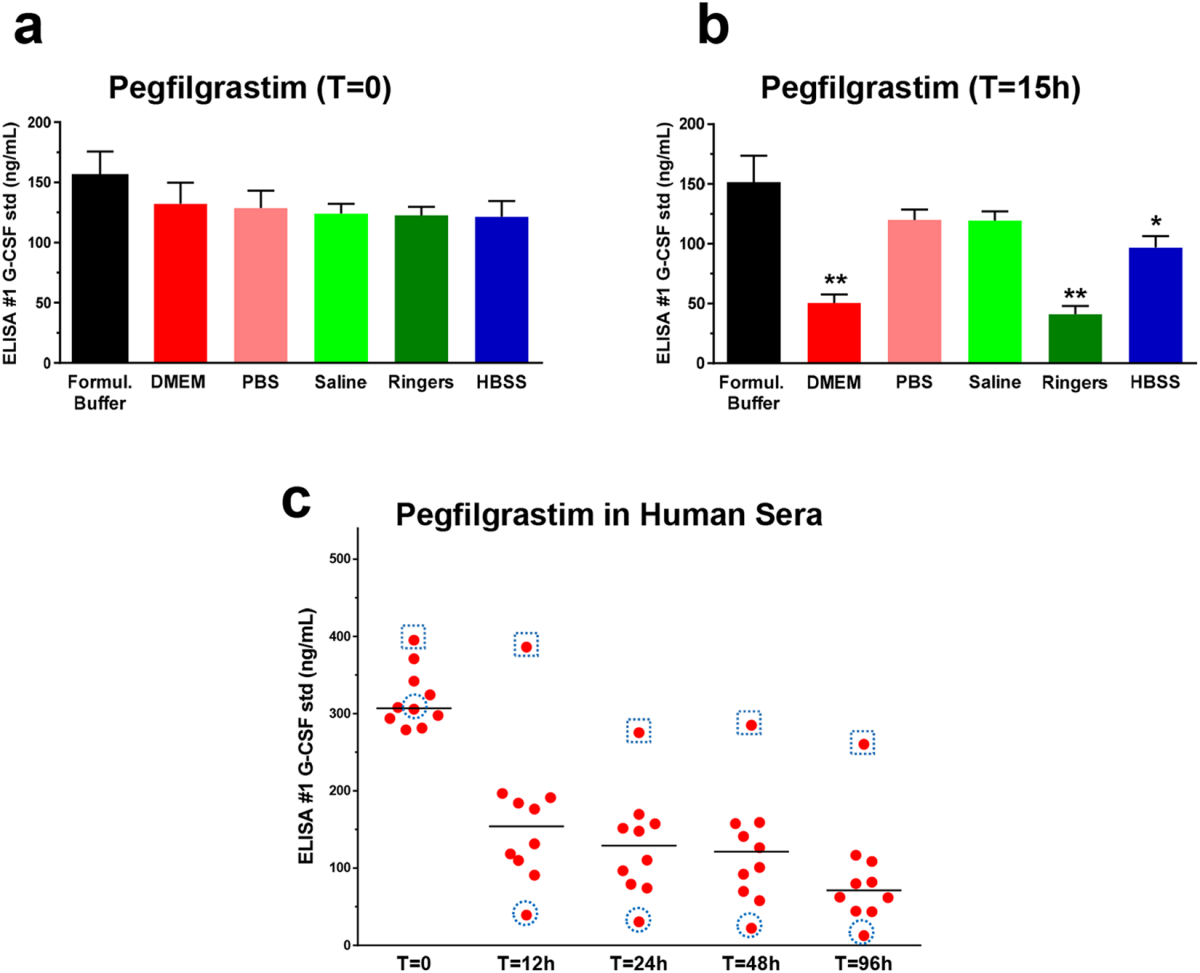
Levels of pegfilgrastim detected in physiological salt buffers and human sera. (**a**,**b**) Pegfilgrastim (200 ng/mL) was spiked into formulation buffer or various physiological salt buffers as shown, and protein levels were measured using a commercial G-CSF ELISA (#1) (**a**) at baseline (T = 0) or (**b**) following 15 h incubation at 37 °C (T = 15 h). Error bars represent the standard deviations generated from three independent sample preparations and measurements. The symbol, *, indicates statistically significant differences between the levels in each solution compared to the level in the formulation buffer (**p < 0.0001, *p < 0.05, Student’s T-test). (**c**) Pegfilgrastim (400 ng/mL) was spiked into 10 individual sera obtained from healthy human volunteers (Donors S1-S10). Pegfilgrastim levels in each sample were measured using commercial ELISA #1 in a time-course experiment and are shown as red dots. Bars represent the means derived from levels detected in 10 individual sera collected at the time points shown. Red dots surrounded by grey squares or circles highlight results from sera from Donors S08 and S10, respectively, whose stability patterns differed greatly and were studied further in experiments shown in Fig. 6b,c.

To monitor whether the detectability of pegfilgrastim is sustained in physiological conditions, pegfilgrastim was diluted at room temperature in a variety of commonly used physiological media or buffers. Following dilution, baseline pegfilgrastim levels determined using a commercial G-CSF ELISA (designated ELISA #1) were slightly lower compared to levels observed in samples of pegfilgrastim diluted in its recommended formulation buffer (10 mM acetate with 5% sorbitol and 0.004% polysorbate 20, pH 4.0^17^), consistent with low surface binding to the microcentrifuge tubes used for studies. Pegfilgrastim levels showed a further time-dependent decline during incubation at 37 °C in some physiological conditions. As shown in Fig. 1b, the detection of pegfilgrastim in DMEM or Ringer’s solution declined approximately 70% from baseline, and a smaller, but statistically significant decline was observed in Hanks Balanced Salt Solution (HBSS). Downward trends were also observed when pegfilgrastim was diluted in PBS or Normal Saline, but the differences were not statistically significant (p = 0.08). Levels remained constant in the manufacturer’s non-physiological formulation buffer.

To determine whether the decline in detectability observed in certain physiological buffer conditions would also be observed in human sera, pegfilgrastim was spiked into sera from 10 individuals (Donors S01-S10) and incubated at 37 °C and monitored by ELISA in a time course experiment. The average levels detected in these samples following 12 h of incubation was ~1/2 that of baseline levels (T = 0; Fig. 1c). As observed in certain balanced salt solutions and media that mimic physiological conditions, there was a time-dependent decline of pegfilgrastim detected using ELISA #1 when the drug was incubated in human sera. By the conclusion of the 4-day experiment, the average detected levels had decreased to <1/3 of baseline, however substantial subject variability was observed, with one outlying serum maintaining ~75% of baseline pegfilgrastim levels.

### Pegfilgrastim forms aggregates in physiological conditions

**Figure 2:**
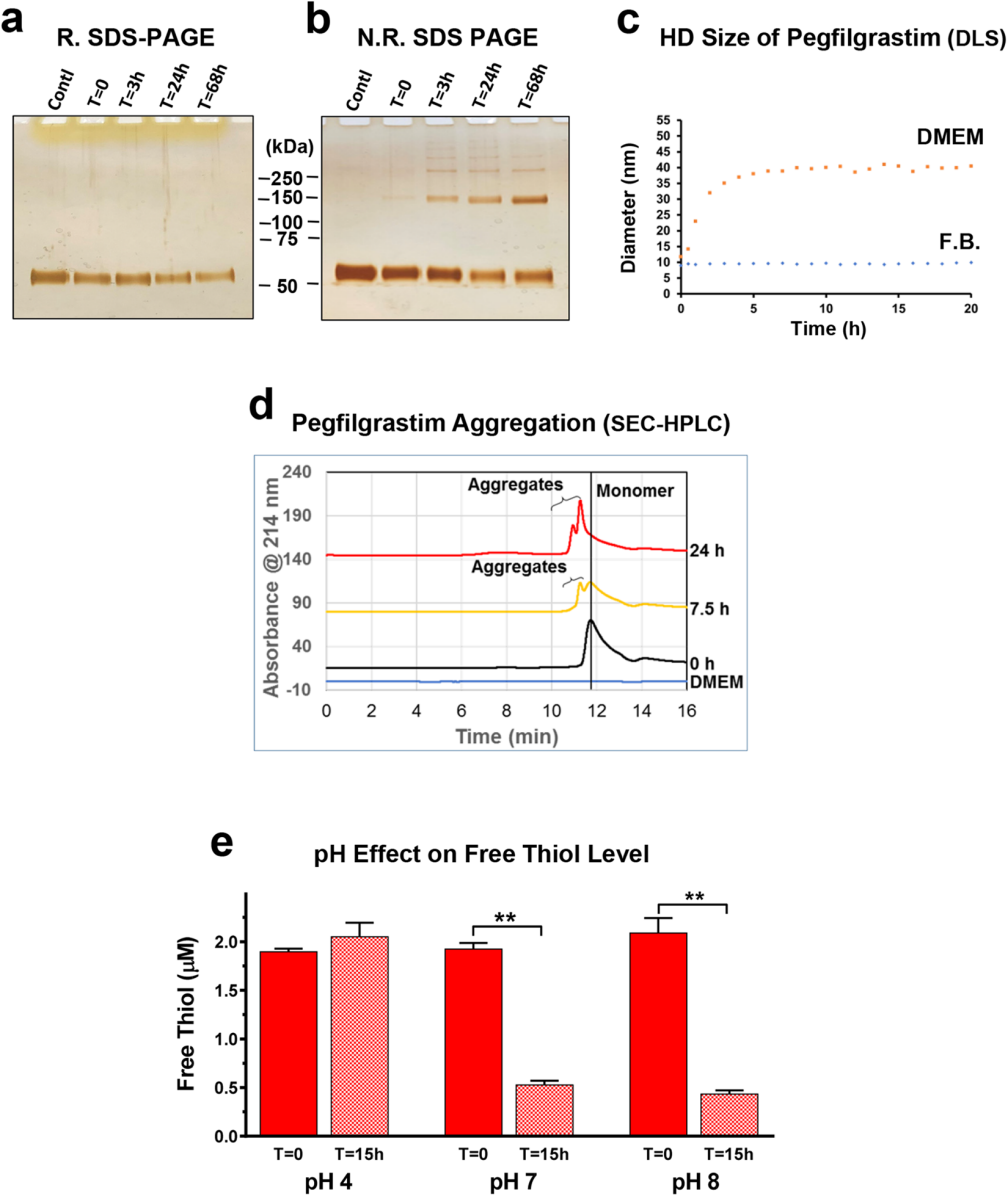
Determinations of the structure of pegfilgrastim in DMEM using orthogonal methods. (**a**,**b**) Pegfilgrastim was reconstituted in formulation buffer (Contl) or DMEM, incubated at 37 °C for the various time points indicated, and analysed by SDS-PAGE in (**a**) reducing or (**b**) non-reducing conditions. (**c)** The hydrodynamic sizes of pegfilgrastim in DMEM or formulation buffer (F.B.) at 37 °C were monitored continuously for 24 h using high throughput dynamic light scattering. (**d**) Pegfilgrastim was diluted in DMEM and incubated at 37 °C. Aggregation was assessed using SEC-HPLC at the indicated time points. (**e**) Pegfilgrastim was diluted to 400 µg/mL with DMEM that pH has previously been titrated to pH 4, pH 7 or pH 8, and the samples were incubated at 37 °C for 15 h. Free thiol levels were measured as shown (n = 4, **p < 0.001 Student’s T-test).

Because a loss of ELISA detectability could be due to structural changes of pegfilgrastim in physiological conditions, as have been previously described at physiological pH^17,18^, the structural integrity of pegfilgrastim was investigated using a series of orthogonal techniques. Proteolytic fragments were not observed during SDS-PAGE analysis performed under reducing conditions (pre-treatment with 50 mM dithiothreitol at 70 °C for 10 minutes), which would indicate that pegfilgrastim does not undergo proteolysis when incubated in DMEM (Fig. 2a). However, when analysed by non-reducing SDS-PAGE, higher molecular weight bands were detected, whose levels increased in a time-dependent manner (Fig. 2b). These findings suggest that covalently-linked disulphide bonds might be involved in the aggregation of pegfilgrastim.

Pegfilgrastim aggregate formation in physiological conditions (DMEM, 37 °C) was also detected by high throughput dynamic light scattering (HT-DLS), which revealed the rapid accumulation of ~40 nm size pegfilgrastim complexes (Fig. 2c). In contrast, the molecular size remained ~10 nm when maintained in formulation buffer. Size exclusion chromatography (SEC) revealed similar increases in aggregate content and depletion of monomer content in DMEM (Fig. 2d). As with results obtained using HT-DLS, SEC analysis showed that the majority of pegfilgrastim aggregates following 24 h in this physiological condition.

Various chemical mechanisms, such as deamidation, reduction, oxidation, and hydrolysis, may be involved in the structural stability of the pegfilgrastim protein moiety^19^. This G-CSF region comprises 175 amino acid residues containing two native disulphide bonds at positions 36–42 and 64–74 and one free cysteine at position 17, which is partially solvent-exposed^20^. The potential involvement of the Cys 17-associated disulphide bond in the aggregation of G-CSF has been of special interest in the field^21^. Previous studies report that G-CSF is prone to form dimeric aggregates under physiological conditions (37 °C, pH 7.0), and aggregated G-CSF contains a population that is disulphide cross-linked^22–24^. Pegylation of G-CSF has a substantial protective effect on its stability^18^, so it was unclear whether pegfilgrastim would share similar characteristics.

We therefore assessed the roles of pH and disulphide cross-linking during the aggregation of pegfilgrastim. To this end, we determined the concentrations of free thiol levels of pegfilgrastim before and after incubation in DMEM that were titrated to pH 4, 7, or 8. As shown in Fig. 2e, free thiol levels were very similar immediately following reconstitution in these media. These results suggest that a rapid, pH-dependent transition involving disulphide shuffling is unlikely. However, following 15 hours of incubation at 37 °C, there was a substantial loss in free thiol levels at pH 7 and pH 8, but not pH 4. This can likely be explained by previously described conformational changes that allow increased accessibility of the free Cys 17^20^ at higher pH levels.

**Figure 3:**
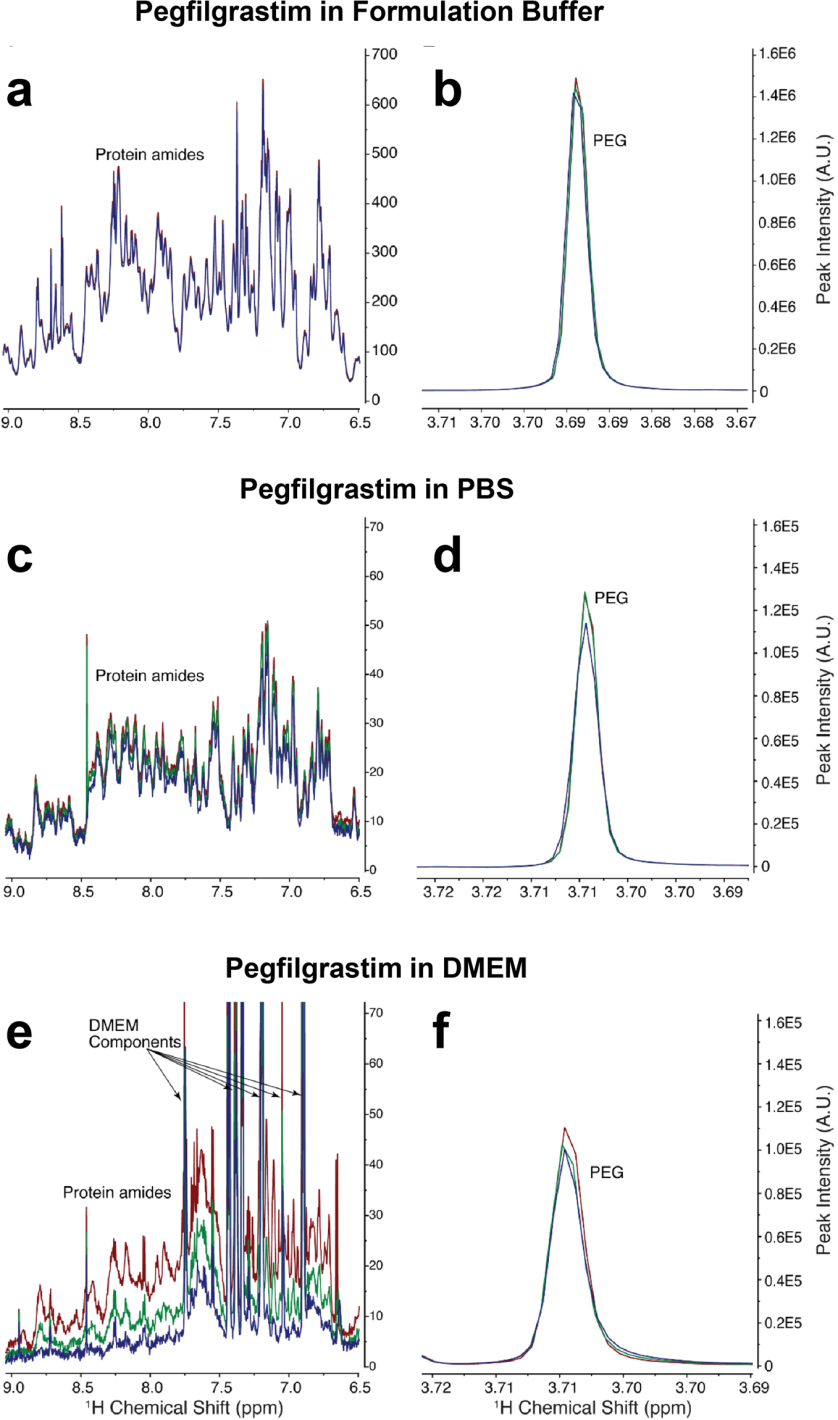
Real-time 1D ^1^H NMR spectra for pegfilgrastim at 37 °C in different buffers (**a**,**b**) formulation, (**c**,**d**) 0.5 × PBS, (**e**,**f**) DMEM. Shown are the overlays of three 1D ^1^H NMR spectra for pegfilgrastim amide region peaks (**a**,**c**,**e**) or PEG peak (**b**, **d** and **f**) in these conditions at 1 h (red), 20 h (green) and 43 h (blue).

We next used 1D ^1^H Nuclear Magnetic Resonance (NMR) to obtain more detailed information regarding the structure of the pegfilgrastim aggregates that form in physiological conditions. In contrast to the stable peak intensities observed for pegfilgrastim maintained in formulation buffer (Figs. 3a,b and S1) or PBS (Fig. 3c,d), NMR studies revealed a time-dependent decrease in the protein peak intensity in DMEM (Figs. 3e and S2). As this drop in the protein peak intensity was accompanied by a stable PEG signal (Fig. 3f), our findings support a model in which the filgrastim moiety of pegfilgrastim, and not the PEG chain, drives aggregation in DMEM. Moreover, the sharp PEG-associated NMR peak suggests that PEG remains highly flexible on the pegfilgrastim surface (Figs. 3f and S1), or water exposed, consistent with a previous study that reported the formation of soluble pegfilgrastim aggregates under physiological conditions^18^. Collectively, these orthogonal assays demonstrate that pegfilgrastim rapidly aggregates in certain physiological conditions, forming soluble particles that initially plateau in size around 40 nm (Fig. 2c). Subsequent structural changes then continue to occur that correspond with further decreased ELISA detectability (Figs. 2b,d,e, 3e, S2b,c).

### Pegfilgrastim detectability in serum stability studies is ELISA-dependent

**Figure 4:**
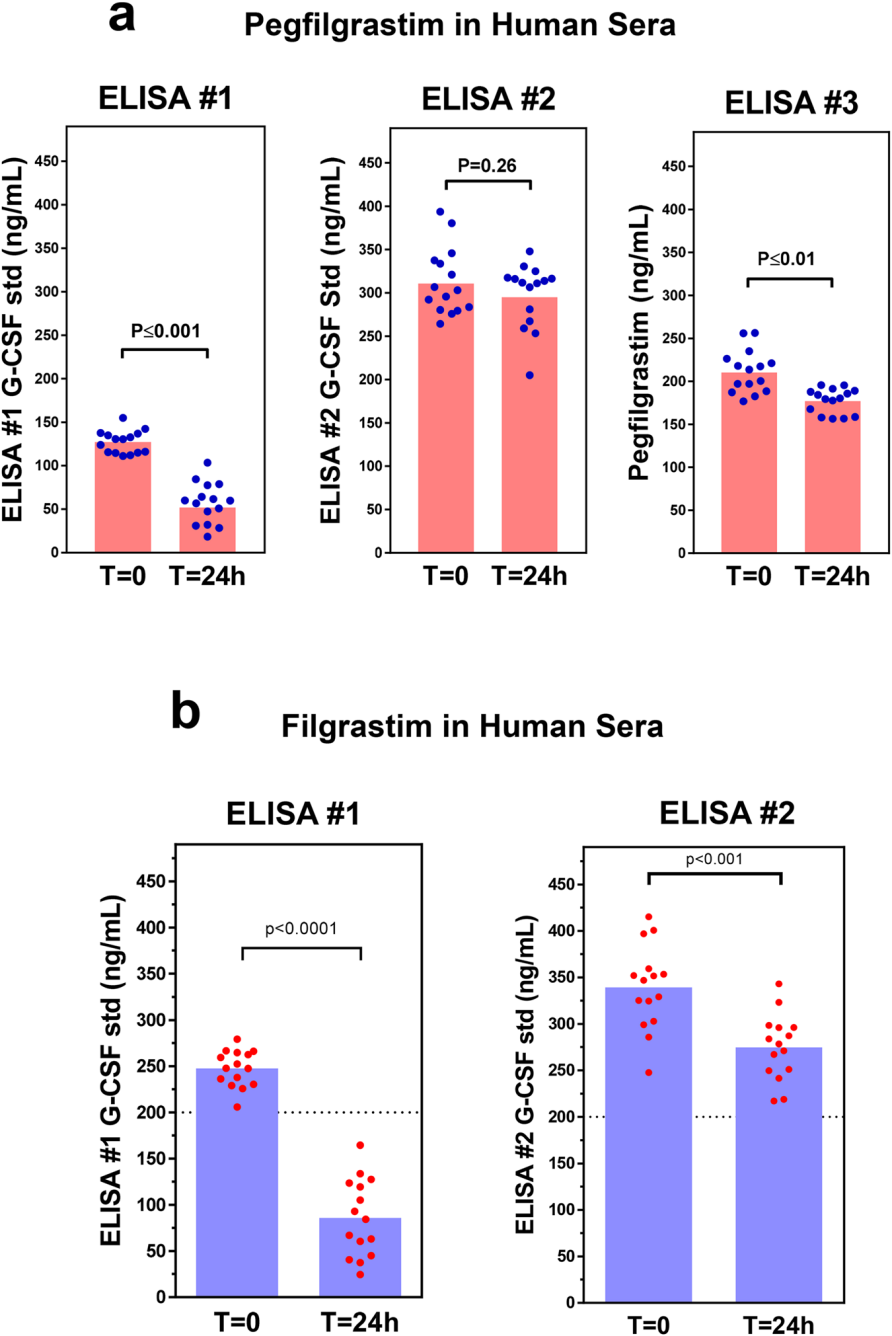
Pegfilgrastim and filgrastim ELISA detection in serum stability studies using various commercial ELISAs. (**a**) Pegfilgrastim (200 ng/mL) was spiked into 15 individual sera obtained from healthy human volunteers (Donors S01-S15). Levels of detection in sera were measured using three commercial ELISA kits (G-CSF ELISA #1, G-CSF ELISA #2, and PEG ELISA #3) at baseline and following 24 h at 37 °C. The G-CSF standards provided by vendors of the two G-CSF ELISA kits (ELISAs #1 and #2) were used to provide relative quantifications for these assays. The standard curve for anti-PEG ELISA assay was generated using an in-house pegfilgrastim standard serially diluted in the assay buffer provided with the commercial kit. Blue dots represent the levels present in each individual sample, and red bars represent means derived from these 15 measurements (p values were calculated using the Student’s T-test). (**b**) Filgrastim (200 ng/mL, dotted line) was spiked into 15 individual sera obtained from healthy human volunteers (Donors S16-30). Levels of filgrastim in human sera were measured using two commercial G-CSF ELISA kits (ELISA #1 and ELISA #2), which utilized different assay-specific quantification standards. Red dots indicate filgrastim levels detected in these 15 individual sera at baseline and following 24 h at 37 °C, and blue bars represent the mean derived from these 15 measurements (p values were derived using the Student’s T-test).

We considered the possibility that commercial ELISAs have varying sensitivities to pegfilgrastim aggregates that form in physiological conditions. To address this question experimentally, pegfilgrastim was spiked into the sera from 15 individuals (Donors S01-S15), and levels at baseline and following 24 h of incubation at 37 °C were then determined using three different commercial ELISAs detecting the G-CSF (ELISAs #1 and #2) or PEG (ELISA #3) regions of pegfilgrastim. As shown in Fig. 4a, the various ELISA formats provided strikingly different results in two respects. First, the determined concentrations varied markedly even at baseline, but this was not unexpected given their assay-specific concentration standards. Secondly, and more importantly, there were differences in the relative degrees of sample stability detected at 37 °C. Levels determined using commercial G-CSF ELISA #1 assay showed a very large decline following incubation in physiological conditions. A commercial PEG ELISA detection kit (#3) showed a statistically significant drop in detection as well, albeit only a ~20% drop on average. In contrast, commercial G-CSF ELISA #2 showed no statistically significant decrease in detected levels, confirming that commercial ELISAs have differing capacities to distinguish monomeric pegfilgrastim from the aggregates that form in physiological conditions.

We next investigated whether the loss of detectability using ELISA #1 was associated with the filgrastim component of pegfilgrastim. Filgrastim was spiked into individual sera (n = 15, Donors S16–S30) and incubated at 37 °C (Fig. 4b). In parallel with the pegfilgrastim results (Fig. 4a), the mean levels of filgrastim detected following 24 h of incubation in sera were much lower compared to baseline levels when ELISA #1 was used for measurements (~35% baseline levels). In contrast, only a modest reduction was observed when ELISA #2 was used (~75% baseline levels). These results paralleled those of pegfilgrastim and are consistent with a model in which the structural changes involving the G-CSF moiety underlies the reduced ELISA#1 detectability of pegfilgrastim in physiological conditions. PEGylation may serve to slow this degradative process, as has been previously proposed^18^.

### The stability of pegfilgrastim is highly variable among individuals

**Figure 5:**
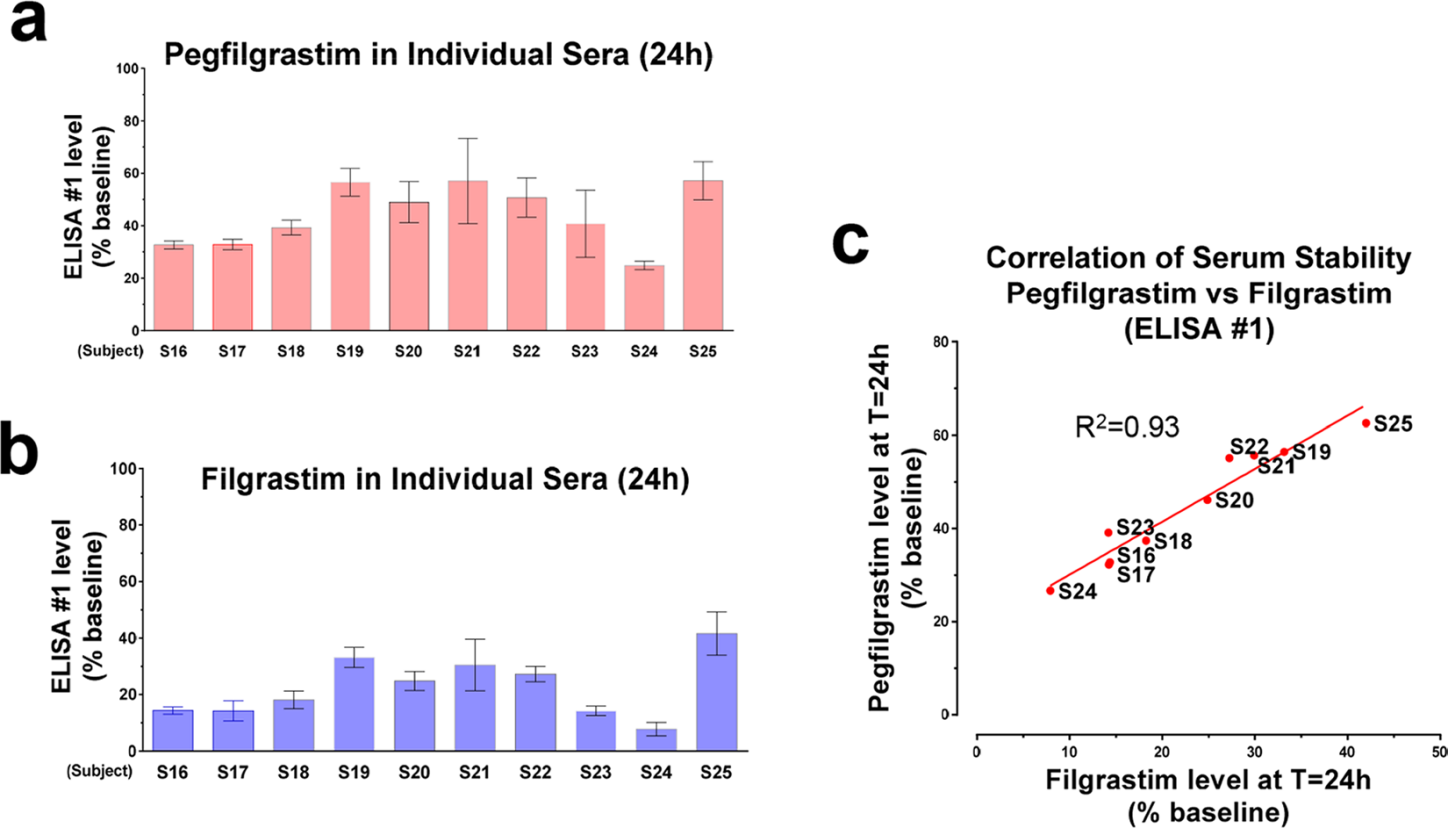
Individual variability of pegfilgrastim and filgrastim in human sera. 200 ng/mL of (**a**) Pegfilgrastim or (**b**) filgrastim was spiked into the sera of 10 individual subjects (Donors S16-S25). The relative drug levels at baseline and following 24 h incubation at 37 °C were assessed using ELISA #1. Data depicted represent mean relative percent detected at T = 24 h vs. baseline levels (n = 4 independent sample preparations, error bars represent s.d.). (**c**) Correlation of the ELISA #1-detected levels of pegfilgrastim and filgrastim spiked into individual sera following 24 h incubation at 37 °C. The data shown in (**a**,**b**) were used for this analysis. The linear regression R squared value was 0.93.

Human donor variability (Fig. 5a), was examined further in stability experiments using individual sera from Donors S16-S25. Drug levels at baseline and following 24 h incubation at 37 °C were measured using ELISA #1 in a total of four independent sample preparations. As is evident in Fig. 5a, there was a marked reduction in detected levels of pegfilgrastim, which was highly donor-dependent. A parallel experiment (Fig. 5b) was performed in which filgrastim (200 ng/mL) was spiked into the same 10 individual sera (S16-25), and the levels of filgrastim at baseline and following 24 h treatment at 37 °C were measured using ELISA #1. Interestingly, the relative stability pattern of pegfilgrastim among the tested individuals closely matched that of filgrastim (Fig. 5a,b). As shown in Fig. 5c, there was a direct correlation between the serum stability of pegfilgrastim and the serum stability of filgrastim (r^2^ = 0.93), supporting the conclusion that the G-CSF moiety is central to the degradation of pegfilgrastim in human serum.

### Pegfilgrastim bioactivity correlates directly with ELISA detectability

**Figure 6:**
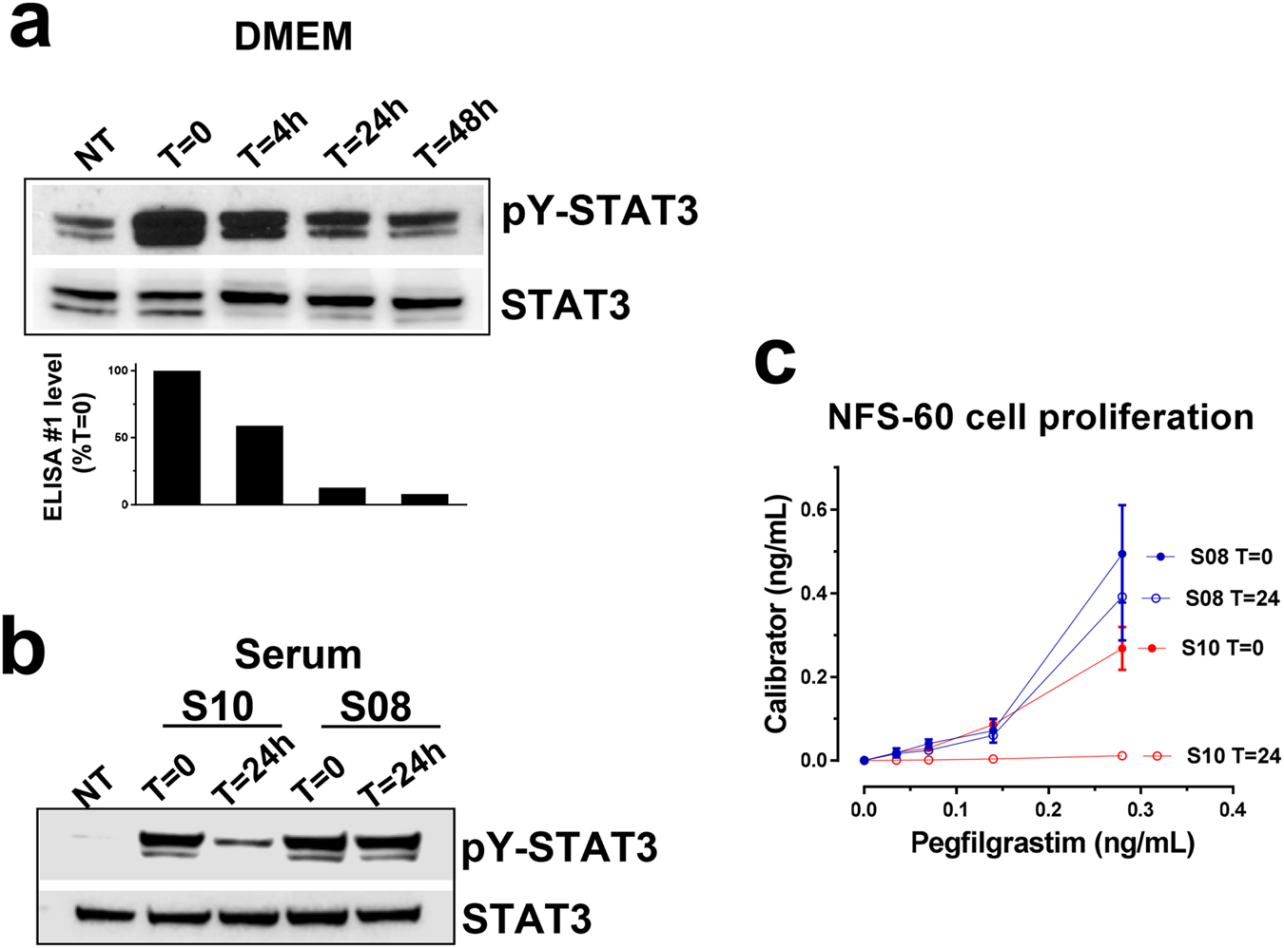
Bioactivity of pegfilgrastim in physiological conditions (**a**) Shown in the upper panel are results from Western blotting experiments demonstrating STAT3 phosphorylation in NFS-60 cells stimulated with or without pegfilgrastim pre-incubated for 0–48 h in DMEM at 37 °C in low protein binding microcentrifuge tubes. Shown in the lower panel are ELISA#1-detetected levels from the pegfilgrastim samples used for the stimulations. (**b**) Previous experiments demonstrated that serum from Donor S08 supported the greatest stability of the ELISA-detected signal, whereas serum from Donor S10 promoted nearly a complete loss of signal following overnight treatment in physiological conditions (Fig. 1c). Shown are Western blotting results demonstrating STAT3 phosphorylation in NFS-60 cells at baseline or following stimulation by pegfilgrastim maintained in these individual sera with or without pre-incubation for 24 h at 37 °C. (**c**) The proliferative capacity of pegfilgrastim following pre-incubation in two different human sera was determined in NFS-60 cells. Proliferation was determined following stimulation by pegfilgrastim maintained in individual donor sera (Donors S08 and S10) with or without pre-incubation for 24 h at 37 °C. Shown are mean proliferation values +/− s.d.

It was also critical to investigate the correlation between the decreased ELISA detectability of pegfilgrastim in physiological conditions and its biological activity to understand the potential implications of these results. To this end, we preincubated pegfilgrastim in DMEM in low protein binding microcentrifuge tubes for varying lengths of time (0–48 h) at 37 °C, which led to a time-dependent decrease in ELISA#1 detectability (Fig. 6a, lower panel). The bioactivity of pegfilgrastim in these samples was then assessed by analysing the capacity of these samples to stimulate the phosphorylation of signal transducer and activator of transcription 3 (STAT3) in NFS-60 cells, a G-CSF receptor-expressing murine cell line derived from a myelogenous leukaemia. Phosphorylation of tyrosine Y705 of STAT3 is the primary consequence of G -CSF-R activation in neutrophil and myeloid lineages^25,26^. The capacity of the pegfilgrastim samples to induce STAT3 phosphorylation decreased with their time of pre-incubation in physiological conditions, paralleling the drop of pegfilgrastim ELISA#1 detectability (Fig. 6a, upper and lower panels).

Next, we examined the correlation of ELISA detectability and bioactivity of pegfilgrastim in serum. Two donors were identified, S08 and S10, whose pegfilgrastim serum stability characteristics differed greatly. Serum from donors S08 and S10 was spiked with 400 ng/mL of pegfilgrastim and incubated at 37 °C. Following 24 h in these conditions, the pegfilgrastim level in Donor S08 serum was ~70% baseline, whereas a <10% baseline level was observed in serum from Donor S10 (Fig. 1c). The pY-STAT3 level stimulated by the S10 sample preincubated for 24 h was markedly lower than that of the corresponding S08 sample, indicating reduced bioactivity in this sample. In contrast, the S08 sample preincubated for 24 h showed no substantial reduction in pY-STAT3 levels, correlating with its retained ELISA detectability in physiological conditions.

In addition, the pegfilgrastim samples incubated in S08 and S10 sera were assessed for their capacity to induce proliferation of NFS-60 cells^27–29^, which are widely used in bioassays for assessing bioactivity of pegfilgrastim and filgrastim. Paralleling STAT3 activation results; the proliferative activity of pegfilgrastim incubated in S10 serum for 24 h was lower than that for corresponding samples incubated in S08 serum (Fig. 6c). Taken together, pegfilgrastim bioactivity, as determined by signal transduction capacity and downstream induction of proliferation, correlated directly with detectability using a commercial ELISA with differential specificity for non-aggregated vs. aggregated pegfilgrastim.

## Discussion

Our results support the conclusion that pegfilgrastim aggregates in certain physiological conditions. This dynamic aggregation process is mediated by physicochemical changes associated with the filgrastim moiety and ultimately leads to irreversible disulphide bond formation. Whether surface binding to microcentrifuge containers accelerates this process is unclear, but the use of low protein binding surfaces in our experiments minimized this factor. The detectability of pegfilgrastim aggregates varies between several commercial G-CSF and PEG ELISA detection kits, at least one of which has served as a development platform for assays for clinical pegfilgrastim concentration determinations^12–14^. Moreover, the formation of inactive pegfilgrastim aggregates that are either directly (physiological buffers) or indirectly (human sera) observed occurs at physiologically relevant concentrations, suggesting these aggregates may impact the interpretation of pegfilgrastim ELISA results *in vitro* or *ex vivo* in clinical PK studies.

The pH of the environment is likely one component underlying the physicochemical basis for the aggregation of filgrastim and pegfilgrastim in physiological conditions. At low pH, filgrastim, a member of the four-helix bundle class of cytokines, is compact, shows a high degree of α-helicity, and is resistant to aggregation^22,30^. However, spectroscopic analysis of filgrastim reveals that distinct conformational changes occur between pH 5 and pH 7.0^22,30,31^, consistent with the change in size of pegfilgrastim that we observed by HT-DLS immediately upon addition to DMEM. Previous published reports indicate that filgrastim aggregates and rapidly precipitates at pH 6.9 and 37 °C^24^. However, these initial studies investigating formation of filgrastim aggregates were performed at high concentrations that are not physiologically relevant. Filgrastim aggregate formation was concentration-dependent, and, thus, it was thought that aggregation would not be a concern at the picomolar range concentrations found in the blood stream^18,22^.

Pegfilgrastim has been proposed to form aggregates via the same degradation pathway as filgrastim. Both have been shown to have almost identical secondary structural transitions, accompanied by the formation of aggregates with nearly identical covalent characteristics^18^. Our finding of highly comparable patterns of human subject variability of filgrastim and pegfilgrastim stability in individual sera further supports a common aggregation pathway for these two molecules. Pegfilgrastim incorporates a PEG moiety, known to increase the solubility and stability of proteins^32^, likely allowing the formation of soluble aggregates under the same conditions that cause G-CSF to precipitate^22,24^. It is also conceivable that the conjugation of PEG to filgrastim decreases its rate of aggregation. Importantly, it should be considered that, in contrast to filgrastim, pegfilgrastim has a prolonged circulating half-life, increasing the likelihood that aggregation may impact its detectability prior to clearance (e.g., by receptor-mediated pathways).

We hypothesize that formation of pegfilgrastim aggregates masks certain epitopes recognized by commercial ELISAs. This would be consistent with the high level of aggregate detection with a commercial PEG ELISA detection kit, as NMR studies indicate that the hydrophilic PEG components in pegfilgrastim aggregates remain directed outward toward the water interface. In this scenario, hidden epitopes would be in unexposed regions in the G-CSF components of the pegfilgrastim aggregates. However, proprietary information regarding the epitopes recognized by the antibodies used in commercial ELISAs is not available to confirm this directly. We are currently conducting studies to evaluate the performance of two of the ELISA assays described in this study according to FDA’s Bioanalytical Method Validation Guidance^33^.

Based on our findings in certain physiological buffers, the conversion of pegfilgrastim in human sera into inactive aggregates was expected. Nevertheless, the level of individual variability in the stability of pegfilgrastim in human sera samples was striking. In this regard, pegfilgrastim has been shown to display considerable subject variability in human PK studies^12–16^. Previously this variability was attributed to variability in cellular uptake, but our data suggest that differences in serum stability may play a large role. Subject variability in the serum stability of pegfilgrastim could represent a confounding factor in the PK studies used to establish PK similarity, supporting advantages for crossover study designs that mitigate this concern.

Although our results elucidate some of the factors that impact the potency and detectability of pegfilgrastim in physiological conditions, future studies are indicated to address remaining questions. For example, the primary mechanism of pegfilgrastim clearance from the circulation has been thought to be neutrophil-mediated clearance via G-CSF-R. This receptor binds pegfilgrastim, and the drug–receptor complex is internalized and degraded inside the cell^34^. Although ELISA results involving serum samples are similar to those with physiological media, we have not directly confirmed that pegfilgrastim forms aggregates *in vivo*. It will be valuable to develop methods to detect and quantify pegfilgrastim aggregates *in vivo* and, if present, investigate their impact on pegfilgrastim bioactivity and detectability. In addition, the human factors in serum that affect pegfilgrastim stability remain to be identified. It will also be important to determine whether certain product variants present in production lots, or the deliberate targeting of quality attributes (e.g., glycosylating the protein), could alter serum stability characteristics. Nevertheless, the data described herein are sufficient to warrant caution regarding the limitations of the interpretation of pegfilgrastim concentrations derived from ELISA-based methods, which have different capacities to differentiate non-aggregated vs. aggregated and biologically active vs. inactive products. Moreover, the impact of individual variability on the stability of protein therapeutics such as pegfilgrastim, highlight the need for a robust PK study design to meet endpoints for biosimilar development.

## Methods

Human serum samples used in this study were acquired from a commercial source (Equitech Enterprises, Inc., Kerrville, Texas), and personal identifying information was not available, so none of the studies was considered human subject research after consultation with a representative from CDER’s institutional review board. The anonymized serum samples were collected by the commercial source from normal adult individuals, with a diverse range of ethnicity and ages of both genders. The serum aliquots were stored at −70 °C and thawed on ice before being used in experiments.

### ELISA assays for pegfilgrastim and filgrastim level measurements

Three commercially sourced enzyme-linked immunosorbent assay (ELISA) kits were used to quantitatively assess levels of pegfilgrastim and filgrastim in sera, media, or physiological balanced salt buffers as follows: (1) ELISA #1 (Human G-CSF Quantikine ELISA Kit, Cat. DCS50, R&D Systems); (2) ELISA #2 (G-CSF Human Instant ELISA™ Kit, BMS2001INST, Invitrogen); and (3) ELISA #3 (PEGylated protein ELISA kit, ADI900-213-0001, Enzo). The assays were performed following the manufacturers’ recommended protocols, modified as indicated. To minimize the interference of surface binding effects, Eppendorf LoBind microcentrifuge tubes (Eppendorf™, # 022431081) were used in experiments involving incubation of low concentrations of pegfilgrastim in protein-free buffers/DMEM. The three commercial ELISA assays were not independently validated as per the FDA’s Bioanalytical Method Validation Guidance^33^. The rh-G-CSF standards provided with the Kits #1 and #2 were used as calibrators for relative quantitation. Serial dilutions of Neulasta (pegfilgrastim) using the dilution buffer provided with kit #3 were used for relative quantitation in the anti-PEG ELISA assay. The optical densities of ELISA plate wells were acquired using a Molecular Device SpectraMax Plus 384 Micro plate reader, and a four-parameter logistic (4-PL) curve-fit was applied for calculating the results.

### NFS-60 cells proliferation and STAT3 activation

Determinations of STAT3 activation were used to assess the bioactivity of the pegfilgrastim samples. m-NSF-60 cells (ATCC^®^-1838™) at a concentration of 5–7 × 10^5^ cell/mL, were stimulated for 30 min with samples containing pegfilgrastim pre-incubated in DMEM or human sera (1 ng/mL final concentration). Total protein cell lysates were prepared using RAPA buffer supplied with Halt™ proteases and phosphatases inhibitors (Thermo #1861280). The protein bands were separated and revealed by standard SDS-PAGE gel and Western Blot technologies. Antibodies used in Western Blot detection were rabbit anti-GCSF (Novus Biologicals, NBP1-89894), mouse anti-STAT3 (pY705) (BD 612357), and STAT3 mAb (9D8) (MA1-13042). The bioactivity of the pegfilgrastim in samples was also assessed using a m-NSF-60 cell proliferation assay. NSF-60 cells (2–3 × 10^5^/mL) grown in complete medium were stimulated by the addition of test samples, followed by continued culture for 48 hours. Cellular proliferation was quantitatively assessed using the WST-1 Colorimetric Assay (Sigma Cat# 11-644-807-001), performed per the manufacturer recommended protocol.

### Reduced and non-reduced SDS-PAGE analysis electrophoresis

Pegfilgrastim was reconstituted in formulation buffer or DMEM to a concentration of 5 μg/mL and maintained at 37 °C for the indicated time periods (up to 68 h). SDS-PAGE analysis was performed using standard techniques and the NuPAGE 4–12% Bis-Tris Gel system (Invitrogen) following the manufacturer suggested protocol. A commercial silver stain assay (Pierce, Cat. 24612) was used to reveal protein bands on the gel.

### SEC-HPLC

To assess changes in quaternary structure, pegfilgrastim was analysed by size exclusion chromatography (SEC) using an Agilent AdvanceBio SEC. 130 Å, 7.8 × 300 mm, 2.7 µm column (cat# PL1180-5350) and 50 mm guard (cat# PL1180-1350) on an Agilent 1260 HPLC. The mobile phase, flow rate, and run time were the same as previously described^27^. Pegfilgrastim (Amgen, Lot# 1063064,) was diluted in formulation buffer according to the package insert to a concentration of 200 µg/mL. Samples were further diluted 1:40 to a final concentration of 5 µg/mL into phenol red-free DMEM (ThermoFisher, cat #31053-028) in a low protein binding microcentrifuge tube (Eppendorf LoBind) and incubated at 37 °C for 0–24 h prior to transfer to an HPLC vial for analysis. SEC-HPLC samples were injected at 5 µg/mL with a fixed injection volume of 100 µL, and the protein was detected by measuring UV absorbance at 214 nm.

### High throughput dynamic light scattering

A DynaPro II Plate Reader HT-DLS instrument from Wyatt Technologies (Santa Barbara CA) was used to evaluate the hydrodynamic size of pegfilgrastim drug product in DMEM. An algorithm-driven high throughput screening method was developed to monitor the size changes of pegfilgrastim maintained in formulation buffer or DMEM at a concentration of 1.25 mg/mL and monitored continuously under physiological temperature conditions^35^. DYNAMICS software from the same vendor was used to record and analyse the light scattering data to determine the hydrodynamic size of the pegfilgrastim in solution.

### Quantitation of free thiol levels

Pegfilgrastim was diluted in DMEM to a final concentration of 400 µg/mL and incubated at 37 °C for the indicated times in Eppendorf LoBind microcentrifuge tubes. The levels of free thiol of pegfilgrastim samples were measured using the commercial Measure-iT Thiol Assay Kit (Molecular Probes, M30550), following the manufacturer’s recommended protocol.

### NMR spectroscopy

To evaluate the structure of pegfilgrastim in formulation buffer, PBS buffer, or DMEM medium, spectra of 1D ^1^H were collected on a Bruker Avance III 850 MHz spectrometer equipped with a QCI cryo-probe. The NMR samples that were analyzed comprised 0.035 mL of pegfilgrastim (Neulasta®), 0.02 mL of D_2_O, and 0.295 mL of formulation buffer, PBS buffer, or DMEM medium. The experimental temperature was 37 °C. The 1D ^1^H NMR experiments were performed using Bruker pulse program *p3919gp* with 128 scans averaged. The experimental time for 1D ^1^H NMR was 6 minutes. Spectra were continuously collected over a time course of 48 h to monitor structural changes. NMR data were processed and analyzed using MestReNova 11.0 software (Mestrelab Research S.L.).

### Statistics

Student’s T-test and linear regression analyses were performed using GraphPad Prism 6 software. The SAS procedure Proc Mixed analysis was applied to determine the subject effect on pegfilgrastim and filgrastim stability in experiments shown in Fig. 5a,b. As illustrated by Eq. (1), *y*_*ij*_ is 100*the ratio of the pegfilgrastim level measured by ELISA at 24 h over the pegfilgrastim level at the baseline for the *i*^*th*^ subject and *j*^*th*^ sample, *y*_*ij0*_ is the pegfilgrastim level at the baseline for the *i*^*th*^ subject and *j*^*th*^ sample, *a*_*i*_ is the *i*^*th*^ subject and is a random effect, and *ε*_*ij*_ is the random error term for the *i*^*th*^ subject and *j*^*th*^ sample. In the mixed effect model (1), we further assumed that *a*_*i*_ is a normal variable with a mean of zero and a variance of ${\sigma }_{a}^{2}$*, ε*_*ij*_ is a normal variable with a mean of zero and a variance of ${\sigma }_{e}^{2}$, and *a*_*i*_ is independent of *ε*_*ij*_.

1 $${y}_{ij}=\mu +{\beta }_{1}\,{y}_{ij0}+{a}_{i}+{\varepsilon }_{ij}$$

Here *I* = 1, 2,…10, and *j = *1, 2, 3, 4.

## Supplementary information

Supplemental Information.
