# Supplementary material for: The ELISA Detectability and Potency of Pegfilgrastim Decrease in Physiological Conditions: Key Roles for Aggregation and Individual Variability
Source: Sci Rep. 2020 Feb 12;10:2476. doi: 10.1038/s41598-020-59346-z (PMC7016140; doi:10.1038/s41598-020-59346-z)

**Supplementary Information**

**The ELISA Detectability and Potency of Pegfilgrastim Decrease in Physiological Conditions: Key Roles for Aggregation and Individual Variability**

Tao Xie^1^, Hui Fang^1^, Weiming Ouyang^1^, Phillip Angart^1^, Meng-Jung Chiang^1^, Ashwinkumar A. Bhirde^1^, Faruk Sheikh^1^, Patrick Lynch^1^, Ankit B. Shah^2^, Sharadrao M. Patil^3^, Kang Chen^3^, Meiyu Shen^4^, Cyrus Agarabi^1^, Raymond P. Donnelly^1^, Kurt Brorson^1^, Sarah J. Schrieber^2^, Kristina E. Howard^2^, Sarah M. Rogstad^3^, David M. Frucht^1^*

^1^Office of Biotechnology Products, Office of Pharmaceutical Quality; Center for Drug Evaluation and Research, U.S. Food and Drug Administration, Silver Spring, Maryland, United States of America

^2^Office of Clinical Pharmacology, Office of Translational Sciences, Center for Drug Evaluation and Research, U.S. Food and Drug Administration, Silver Spring, Maryland, United States of America

^3^Office of Testing and Research, Office of Pharmaceutical Quality; Center for Drug Evaluation and Research, U.S. Food and Drug Administration, Silver Spring, Maryland, United States of America

^4^ Office of Biostatistics, Office of Translational Sciences, Center for Drug Evaluation and Research, U.S. Food and Drug Administration, Silver Spring, Maryland, United States of America

**Supplemental Figure 1** 1D ^1^H NMR spectra of the pegfilgrastim drug formulation. The aliphatic region, 0-2.0 ppm, is free of major excipients (e.g., sorbitol and acetate).


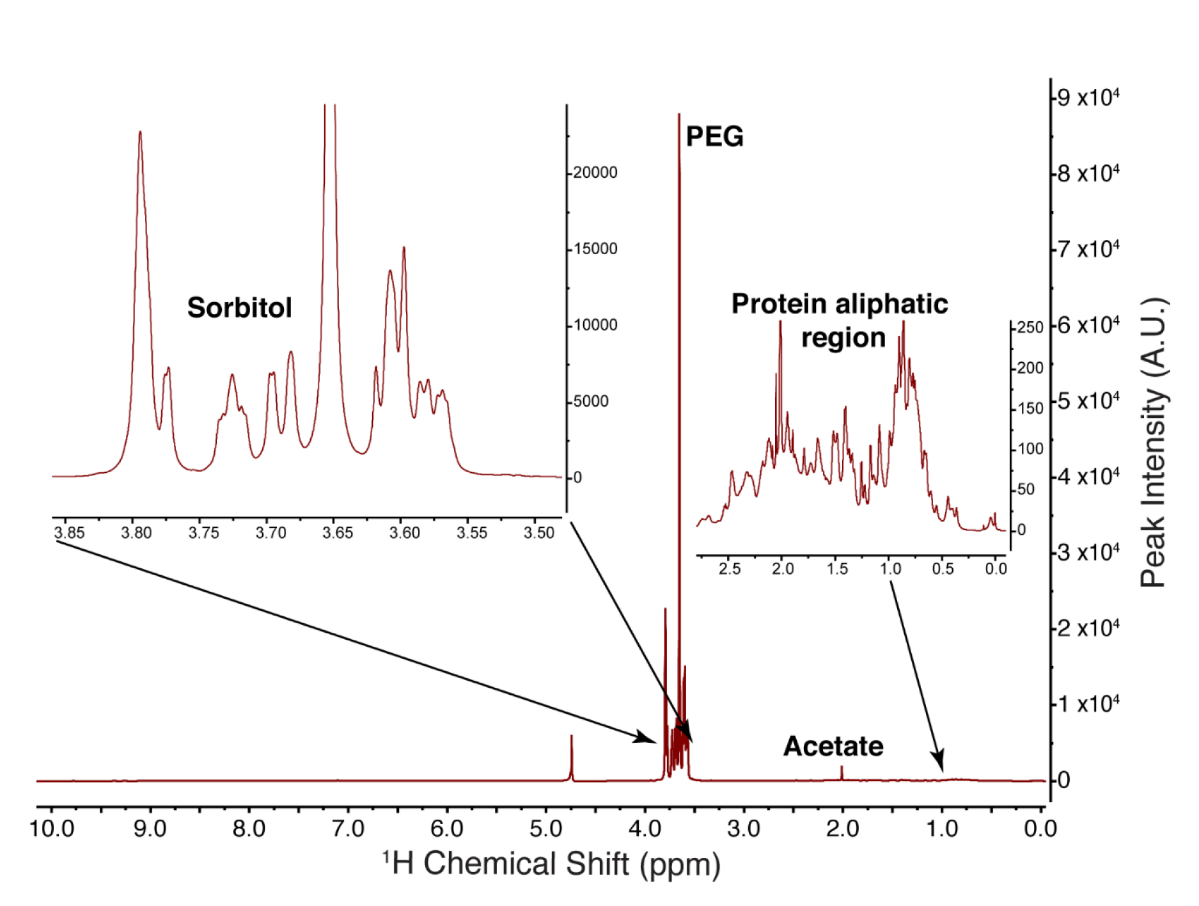


**Supplemental Figure 2** 1D ^1^H NMR study of pegfilgrastim in DMEM buffer at 37 ºC. Shown are the full spectra (A) and zoomed-in spectra (B) of the aliphatic region. The overlay of three spectra shown in (B) show 1 h (red), 20 h (green) and 43 h (blue) time points. The integration of the chemical NMR shift region from 0.60-0.89 ppm allowed the evaluation of peg-filgrastim aggregation kinetics shown in (C).


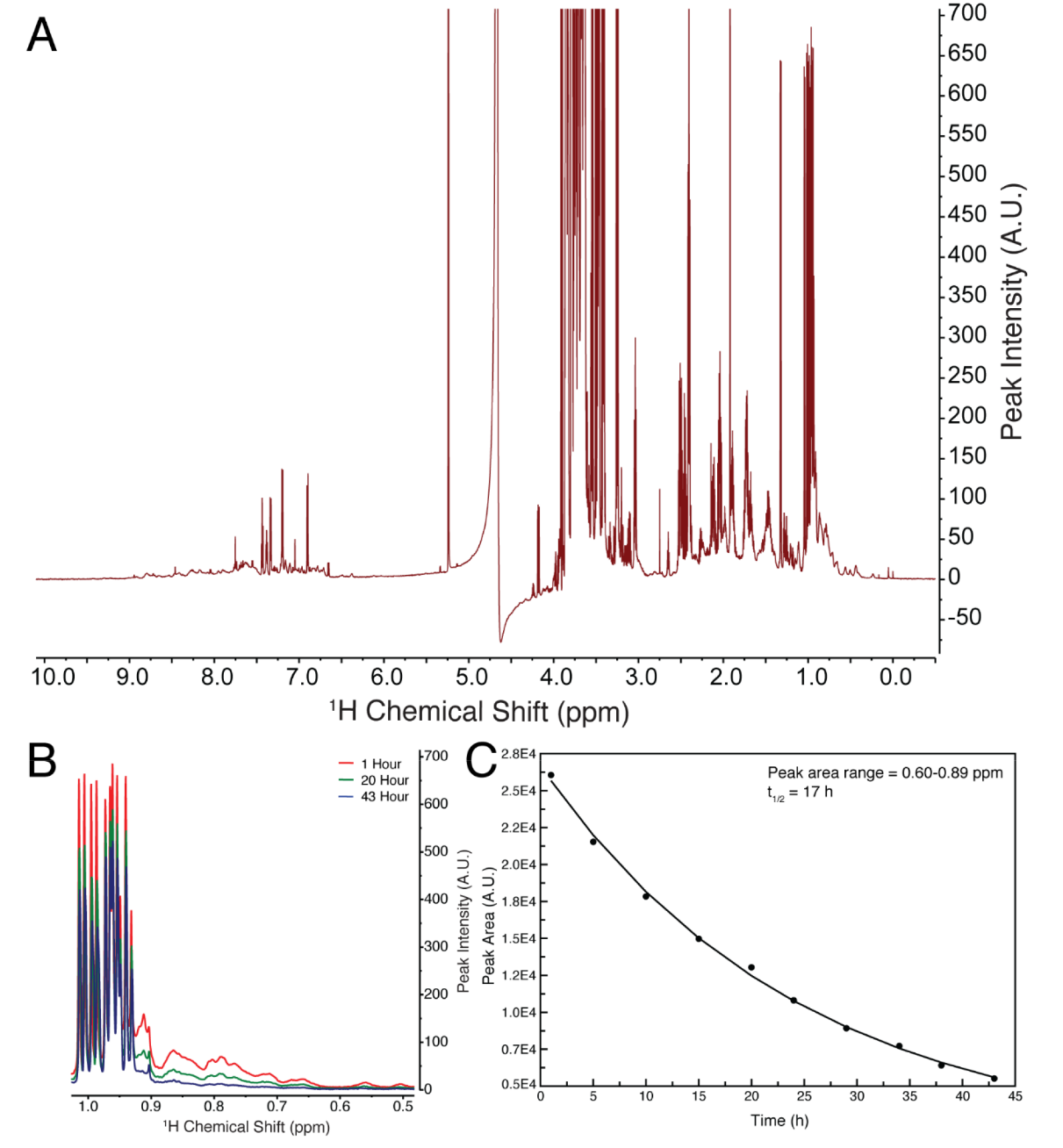

Supplement: Supplementary file 1 — Supplemental Information. [file 41598_2020_59346_MOESM1_ESM.docx]
